# A Systematic Comparison between the Ethereum and Hyperledger Fabric Blockchain Platforms for Attribute-Based Access Control in Smart Home IoT Environments

**DOI:** 10.3390/s23167046

**Published:** 2023-08-09

**Authors:** Stefan Pancari, Anik Rashid, Jason Zheng, Shirali Patel, Yi Wang, Jian Fu

**Affiliations:** 1 Department of Electrical and Computer Engineering, Manhattan College, Riverdale, NY 10471, USA; spancari01@manhattan.edu (S.P.);; 2 Department of Electrical Engineering and Computer Science, Alabama A&M University, Huntsville, AL 35762, USA

**Keywords:** blockchain, Ethereum, Hyperledger Fabric, IoT

## Abstract

Despite the lack of blockchain systems being utilized in modern IoT environments, the prevalence of blockchain technology is increasing, due to its high level of security and accountability. The integration of blockchain technology and access control in a decentralized system for smart home networks is a promising solution to this issue. This paper compares the implementation of attribute-based access control (ABAC) with two popular blockchain platforms, Ethereum and Hyperledger Fabric, for a smart home internet of things (IoT) environment. We present a comprehensive summary of access-control and blockchain-access-control methods, to provide the necessary background for this study. Additionally, we present an original ABAC smart contract for Ethereum, and the modification of a pre-existing Hyperledger Fabric ABAC smart contract, for this comparison. Through the simulation of both implementations, the advantages and limitations will be considered, to determine which is better suited for a smart home IoT environment.

## 1. Introduction

Technological advancements have allowed for the integration of the internet of things (IoT) with home and residential buildings, presenting an advanced method of increasing the usability, security, and quality of life of the user [1]. This is called a smart home network, which is a home equipped with various devices, such as lighting, heating, and monitoring systems that smartphones or computers on the network can control [2]. There are an abundance of advantages that a smart home system provides for the user; however, with all advances in technology, there are a concerns regarding balancing data security and the privacy of the user on the network [2].

To address these security concerns, a centralized structure can be used, but this comes with a risk of being vulnerable to cyberattacks. For this reason, access-control methods can prevent unauthorized users from accessing network resources and data. One simple technique for implementing access control [3,4] is to use a central authority, with a central server [2]. However, this approach also presents a major system vulnerability. The entire network is compromised if the central server fails due to natural factors or cyber-attacks [1,5].

A distributed access-control network is used to overcome the shortcomings of a more traditional centralized network. This replaces the single server with multiple nodes to carry out the access-control system [2,6]. This method is more capable of withstanding cyber-attacks, as well as preventing unauthorized users from gaining access to network resources. One system that can provide this distributed access-control network is the integration of blockchain technology. This technique has opened up a wide variety of possibilities for securing all types of networks, especially in a smart home environment.

Due to its decentralized modular architecture, blockchain technology is a system used for storing data that is far more challenging to hack or alter [7]. A blockchain is a linked data structure that sequentially combines blocks of data and information. The system records the blocks in an encrypted form, as a distributed ledger that cannot be changed or tampered with [8]. Smart contracts [9] are used as a part of the blockchain, and are unique addresses to which end users can address transactions. As a part of smart contracts, transactions that interact with assets are defined [10]. A blockchain uses a distributed networking system of machines that replicate and create a chain of data. This chain of data is considered a ledger, with each of these forming the basis of a block. Smart contracts allow users to interact with ledgers [5,11].

A blockchain is made up of different blocks, each containing transactional information. Multiple blocks can be connected to form a chain, hence the name blockchain. When these blocks are chained together, it makes it challenging to alter data on one specific block, increasing the security of the system [5]. Each block contains information from the previous block, reinforcing the integrity of the data and of the overall blockchain system [12]. Additionally, each block is propagated within the network, allowing each machine to view the chain and all of its data, allowing for multiple verifications to take place [5]. This further ensures the authenticity of data and the integrity of the blockchain. The most common example of a blockchain in use would be in cryptocurrencies, such as Bitcoin and Ethereum [5].

Two of the most widely used open-sourced blockchain platforms are Ethereum and Hyperledger Fabric [13]. They provide a secure method of implementing a decentralized network in a transparent and programmable way. For this reason, Ethereum and Hyperledger Fabric will be the main blockchain platforms discussed in this review paper [14].

Ethereum is a popular blockchain platform used to build public and private blockchain networks. Through an analysis of the performance of this platform, regarding the security response time and the accuracy of the overall system, Ethereum can be compared to other centralized networks [15]. Studies show that the proposed network architecture outperforms the traditional centralized architecture by having a better accuracy and lower response time [16]. Blockchain-based architecture allows for a minimization of the breaches in confidentiality and integrity, and authentication issues in the heterogeneous IoT and centralized gateways, that are present in many security systems. There are possible security issues with the Ethereum blockchain; however, they can be avoided through the consideration of these vulnerabilities during the development of a smart contract [15].

Hyperledger Fabric is a popular blockchain implementation tool that is hosted by the Linux Foundation [5]. It has a modular architecture that is beneficial for customizing private networks. For example, it supports the use of consensus, membership, and database blockchain layers [2,17,18], which allows for a wide range of possibilities, especially for access-control security techniques [19]. In fact, it is possible to build security measures based on member’s attributes using Hyperledger Fabric, which is why it is a popular choice for attribute-based access control (ABAC) [5,17,20]. However, it is not possible to implement a flexible or changing ABAC mechanism, because the parameters and permissions must be predefined on the network [18,21].

Smart contracts allow an automated program to be deployed on a blockchain network, in the form of encoded logic [5]. Developers use basic languages, such as node.js and Java, instead of those like Solidity, to code smart contracts. As a result, developers do not waste time learning a new language, and can create, update, and query device information from the ledger, by submitting transactions to the smart contract [22]. Access control is achieved using smart contracts, especially on an Ethereum platform [2,15].

A blockchain typically adopts a peer-to-peer trading system when it comes to buying and selling cryptocurrencies directly with one another. This makes a blockchain a considerably more secure system compared to centralized networks. Centralized security structures render systems more vulnerable, because the integrity, certification, and availability are compromised. Security threats and vulnerabilities increase with the spread of an IoT system structure: data forgery, tampering access to unauthorized devices, and incorrect device controls. The decentralized structure of a blockchain uses digital ledgers to record transactions and store data across the peer-to-peer network.

This paper presents a comparative study of a decentralized access-control system [23] that employs ABAC and blockchain technology under smart home IoT environments [24]. This will be implemented using a physical Ethereum private network and a simulated smart contract. A Hyperledger Fabric network will also be simulated, to explore a novel comparison of the implementation of ABAC in these two popular platforms [19,25]. The study explores a previously unexplored research direction, by comparing ABAC implementation in Ethereum and Hyperledger Fabric. This offers original advancements in smart contract design and modification, to achieve effective access control in smart home IoT environments.

The contributions of this paper are as follows:

- Firstly, the work compares ABAC implementation in both Ethereum and Hyperledger Fabric platforms.
- A comprehensive summary of access-control and blockchain-specific access-control methods.
- Development of an original ABAC smart contract for the Ethereum platform, without the use of a pre-existing codebase or template.
- Modification of the pre-existing Hyperledger Fabric ABAC smart contract, to be better suited for this comparison.

## 2. Related Work

This section will present some relevant work in this field of study. The main contributions of the studies will be discussed, as well as their shortcomings, to display the importance of the work performed in this paper.

Data security and privacy in IoT devices connected in a smart home environment comprise one of the highest priorities of the system. These IoT devices alone are constantly being exposed to various attacks, and lack the features to defend themselves accordingly. For this reason, many works have been carried out to address this issue, proposing different access-control methods to ensure the security and privacy of devices connected to the network. In this section, some related works will be discussed, to introduce previous implementations of access-control methods, along with the blockchain platform used [26]. Table 1 serves to summarize the findings of this section, by listing the access-control method presented by the authors, and the blockchain implementation platform used, as well as the advantages and limitations of the access-control method used in their work [27].

Kumar et al. [6] built a blockchain-based healthcare network that uses an enhanced Bell–LaPadula model to classify different peers and transactions on the network, with different security levels. This enhancement to the Bell–LaPadula model used discretionary access control (DAC) and mandatory access control (MAC) [6]. DAC manages the established MAC permissions, to provide more flexibility in changing the access-control policies at any time. This model was constructed on the Hyperledger Fabric platform, with 35 peers on the network. The goal was to reduce the scalability issues in the blockchain network, which was successfully accomplished. However, this system is complicated by the use of the MAC, DAC, and Bell–LaPadula models [6] to implement access control. Each of these models has its own limitations, which are mitigated through the integration of other models into the system. However, this makes the system complicated to implement and maintain.

In the system presented by Cruz et al. [28], a role-based access-control (RBAC) framework was built, using smart contracts deployed on an Ethereum blockchain platform [29]. All user–role identifications and assignments are contained in the smart contract, which is then deployed on the blockchain. The system provides a secure and efficient way to define the user–role assignments, and verify the user’s role. Personalization and approval are automatically included in the system, as well [28]. However, due to the nature of RBAC, the role of each user must be predefined before the implementation of this type of access control [30]. In addition, this system is only useful in an organization or a company, where everyone’s individual roles are clearly defined. Even then, it can be a challenge, and can still pose difficulties when roles undergo changes within the company or network.

In a work carried out by Qashlan et al. [31], they proposed the extension of their earlier work [31], in which they presented a lightweight Ethereum blockchain-based multi-tier edge-smart home architecture. Every home in the framework has its own blockchain miners, along with smart contracts being used to ensure the automated enforcement of rules and policies to regulate the IoT devices [31]. This enforcement on the framework uses the attribute-based control (ABAC) approach to enforce rules. The extension of this work proposed the incorporation of cloud servers to increase the storage and analysis of IoT smart home device data [2]. The shortcoming of this experiment was the lack of testing to find the balance between the accuracy and the privacy of the data being transferred. The increase in privacy added more noise, which threatened the readability and accuracy of the data [31]. Despite this, we believe that ABAC is considered the most suitable access-control model for IoT applications. The model allows for dynamic and fine-grained access control, based on attributes, such as the user identity, device type, location, and other contextual information. ABAC can handle complex, dynamic, and heterogenous environments, for which traditional access-control models may not suffice.

**Table 1:** Access-control methods using Hyperledger Fabric and Ethereum for blockchain implementations, and their advantages and limitations.

Method	Advantages	Limitations	Blockchain Implementation Platforms
Discretionary Access Control (DAC) [6]	Used to build a more flexible access-control policy; dynamically change the policy at any time for different subjects and clearance levels	Needs to work with other methods to have a fully functional model	Hyperledger Fabric
Mandatory Access Control (MAC) [6]	Enforces control over peers and resources for better security; suitable if you require a mechanism where the permissions are non-transferable	Lacks the flexibility of other models	Hyperledger Fabric
Role-Based Access Control (RBAC) [28]	Well-suited framework for organizations; versatile framework; can be implemented with smart contracts, to make a more reliable access-control method	Not inherently trans-organizational; without verifying roles, this method is insecure and unreliable	Ethereum
Attribute-Based Access Control (ABAC) [31]	Is more suited for scenarios where the number of roles is increasing; users directly apply the subjects’ attributes, resources, and environmental properties; reduces the number of rule/role updates required	Requires access to a description of the field attributes and the definition of the attributes across many fields	Ethereum
Attribute-Based Access Control (ABAC) [31]	Modular network structure; supports component pluggability for consensus, membership, and database layers	Not suitable for flexible or dynamic applications; permissions must be defined in advance	Hyperledger Fabric

## 3. Systematic Approach

The systematic approach for this review paper is broken down into three phases: planning, research development, and documenting. The research development phase has its own respective subsection phases, the literature review and the experimental process. These three phases were followed in order to ensure the accurate collection of information, as well as a cohesive article. All of the information outside of the experimental results was found via the performance of a literature search in scientific databases. Many of these papers were found under the publisher IEEE, as well as MDPI, Springer, and other academic publishers. During this literature search, we looked for articles that performed experiments of reviews that analyzed blockchain system platforms. These articles supplied information for qualitative research, as well as experiments that can be replicated. However, it was necessary to narrow down the papers to a specific blockchain application, due to the large number of topics found. For this reason, the papers were filtered by the following criteria: they must use either the Ethereum or Hyperledger Fabric blockchain network, must relate to the IoT in a smart home environment, and must implement ABAC.

**Figure 1:**
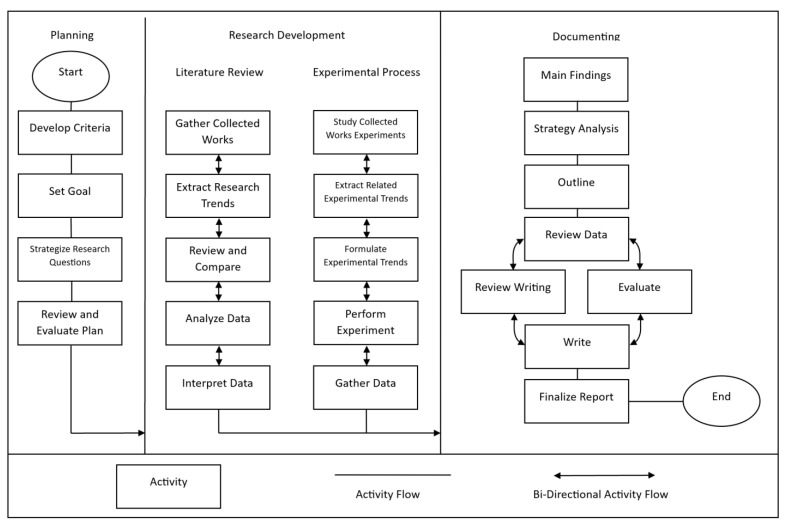
Systematic Approach.

Below is a diagram that shows the methodology in the form of flowcharts (Figure 1). Each phase is labeled respectively, along with the steps that were performed during that process. Each activity box represents an action that was performed, and the activity flow and bi-directional activity flow lines can be followed, so that the reader can understand each phase. A bi-directional activity flow line indicates that the adjacent activities were repeated as many times as necessary. Each phase is explained in more detail below.

### 3.1. Planning

When strategizing the approach to organizing this paper, we set certain criteria for what needed to be discussed. We were able to set the foundations of the paper by providing an overview of the IoT, smart home networks, Ethereum, smart contracts, and Hyperledger Fabric. Additionally, we introduce the diverse forms of access controls set within these systems, such as DAC, MAC, RBAC, and ABAC, within the related works. In this paper, we will mostly focus on the ABAC methods, especially within the Ethereum and Hyperledger Fabric networks [32]. Our planned research goal is to explain what a blockchain is, its architecture, and how it performs, especially in relation to the Ethereum and Hyperledger Fabric blockchain networks that are related to the IoT and ABAC. In the planning phase, we will continue to research questions, implement these questions in real-time testing, and provide information to users, to delve deeper into the comparisons between blockchain networks [18].

### 3.2. Research Development

The research development phase is broken down into two sections: the literature review, and the experimental process. This is because, in this paper, previous research was studied, to form a comprehensive review of the state of the Ethereum and Hyperledger Fabric blockchains with ABAC in an IoT setting [21]. Next, to perform a more thorough review, an experimental setup was conducted, to get a better understanding of how this concept is implemented into a system [32].

#### 3.2.1. Literature Review

During research development, it is crucial to discuss factors related to blockchains, especially within Ethereum, Hyperledger Fabric, the IoT, and ABAC. The sources collected and discussed include smart contracts, and the different programming languages related to blockchains (i.e., Solidity, node.js, Java) [32]. Discussions on diverse access controls within Hyperledger Fabric are also analyzed, so that readers can have a better understanding when interpreting the rest of the paper. We perform qualitative research into where factors accompanying blockchain technology are discussed, specifically within Hyperledger Fabric, Ethereum, the IoT, and ABAC [33]. Additionally, quantitative research is included throughout the duration of this paper, which is more experimental and implementation based. Through proper evaluation, and inferences from the selected papers, visual graphs and tables were created, to ensure that readers can recognize patterns, and broaden their understanding of the blockchain within Ethereum, Hyperledger Fabric, the IoT, and ABAC [34]. We divide this paper into four main sections, in which these topics are discussed in more depth.

#### 3.2.2. Experimental Process

In this phase of the article, the experimental setup is explained in detail, to show how the test networks are set up, and what purpose they serve. The Ethereum private blockchain network is constructed and demonstrated with working ABAC, to secure the network using access control. Likewise, a Hyperledger Fabric network of similar characteristics was also built with ABAC, to perform a review of the similarities and limitations of these two networks. Both qualitative and quantitative data were collected in this section, for the evaluation section of this paper.

### 3.3. Documentation

In this final phase, the results from the literature review and experimental results are composed in such a way as to form a conclusion. This includes organizing the data in their respective sections, and adding documentation to the article, as well as making tables to show and support any conclusions that are made.

## 4. Materials and Methods

**Figure 2:**
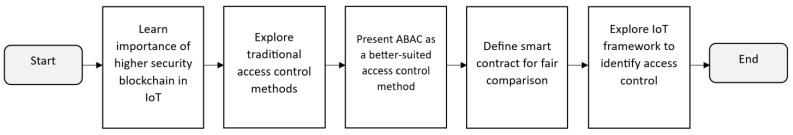
Flowchart to demonstrate section cohesion, to provide adequate background material to perform the comparison.

This section will present the relevant information gathered in the literature review, to conduct a fair and comprehensive comparison between the implementation of ABAC on the Ethereum and Hyperledger Fabric platforms. Figure 2 has been constructed to display the essential steps taken in this section to present relevant information, so that the comprehensive comparison can be made [35]. Each action in the flow chart represents a subsection in this portion of the paper, which will be briefly explained below, to emphasize the importance of this material in the comparison being made.

Firstly, the relevance of the IoT will be discussed, along with the importance of increasing the security of blockchain platforms. Next, traditional access-control methods are researched and presented as a way to increase security, but are ultimately concluded to not be well suited for the secure implementation of access control in a blockchain IoT environment. Then, ABAC will be presented as a better-suited access-control method to be selected for this comparison between Ethereum and Hyperledger Fabric. Next, the term smart contract will be defined, due to the number of times it will be mentioned in this paper, and to ensure that a strong foundation is made for a fair comparison. Lastly, the access-control IoT framework will be provided, to show the various devices and components, to identify the specific access-control requirements to inform our evaluation of the suitability of Ethereum and Hyperledger Fabric for IoT access control.

### 4.1. Importance of Blockchain-Based Access Control in the IoT

**Figure 3:**
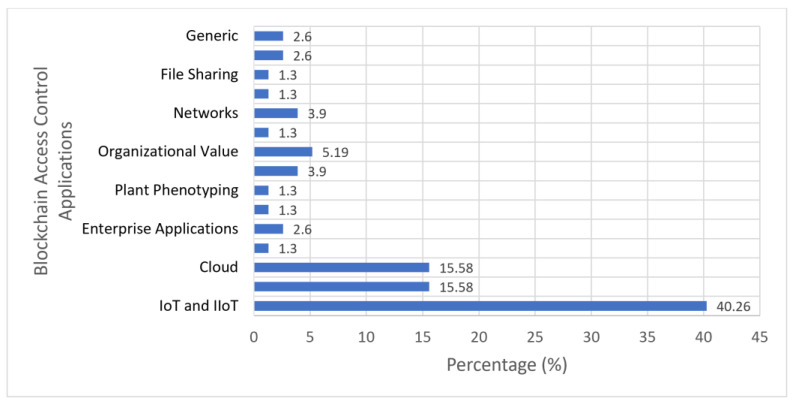
Blockchain-Based Access-Control Applications.

Blockchains were developed to help solve modern-day issues in the industrial and business world. The main purpose of a blockchain is to serve as distributed and immutable storage for access-control policies [19]. This technology is applied to many fields of study, the most significant being the IoT. A graph has been constructed to show all the application domains for blockchain-based access [36] control (Figure 3). This graph was constructed through the adaption of data collected by Dar et al. [37], to show the relevance of conducting a study on the IoT. From studying the graph, we conclude that the IoT and IIoT (Industry Internet of Things) account for about 40% of the entire blockchain application domain. Healthcare [38] and cloud applications [39] are the second most significant portion of blockchain applications, but still fall nowhere close to the IoT. This shows the importance of securing blockchain platforms with access control for the future of the IoT [33].

To provide support for IoT blockchain access control, many industrial and blockchain platforms have surfaced, including Hyperledger Fabric and Ethereum [18]. There lies a different realm of access-control methods affiliated with blockchains, smart contracts, and the IoT. Although there are many access-control methods, only attribute-based access control is sufficient for a blockchain network. The next sections will explore access-control techniques, to present why most are not suitable for blockchain IoT applications, and why ABAC was chosen for this comparison between the Ethereum and Hyperledger Fabric platforms.

### 4.2. Access Control—An Explanation of Traditional Access Control

Access control is a security technique that limits the activities of legitimate users, by regulating their access to certain resources. This regulation is based on company policies, security procedures, and the level of confidentiality the data holds [37]. Access control typically works in conjunction with other security services within systems, to provide optimal security within an environment.

Traditional access control is widely used by companies/organizations to constrict certain actions from being performed, by inhibiting certain actions and operations from being carried out. There are four types of traditional access control: mandatory access control (MAC), role-based access control (RBAC), discretionary access control (DAC), and rule-based access control [38,40]. A brief description of each access control will be discussed, to benefit the reader, and to show why these types of access control are not suitable for a blockchain IoT environment [41]. This will present an explanation as to why these traditional access-control methods are not used for such an environment, and why they are not considered in the comparison between the Ethereum and Hyperledger Fabric access-control networks. A table is provided at the end of this section (Table 2), offering a brief summary of the traditional access-control methods presented, and explaining their unsuitability for blockchain-based IoT smart home systems.

A. Mandatory access control (MAC) is a type of access control where end users are unable to alter information when given access to a certain file system. Individuals that are given access to, and permission for, security controls are restricted from making changes. System administrators or resource owners only have the authority to grant and deny access to resource objects within security controls. MAC systems are able to define access policies through the use of security labels [8]. Its criteria are also pre-established by system administrators, and are usually imposed by operating systems within government and military facilities. MAC operates by organizing and classifying each file system object within resource objects in a file system [42]. When using MAC, users and devices have already-set clearance levels and classifications. Classifications can be distinguished between the following three categories: Confidential, Secret, and Top Secret. Security kernels are implemented and used within MAC [42]. Security kernels are made up of hardware, software, and firmware components. Security kernels serve as the center point of computers, and are the most trusted part of a computer. This was formally referred to as the TCB or trusted computer base. Security kernels check a user’s authority and credentials, before granting access to a resource [40]. MAC is the most secure access control, but it takes a significant amount of time to set it up, and to ensure the resource objects and user information are up to date [42], meaning it is not ideal for many blockchain applications.
B. Role-based access control (RBAC) determines who is authorized to access certain controls, based on his or her role within an organization. Levels of access increase based on authority, responsibility, and how important the role is. Only certain access controls are given to complete a certain task. Access controls are narrowed by restricting behaviors, such as creating files, modifying files, and viewing files. The lower importance and authority given to a user within a company, the fewer access controls an individual will have [40]. This access-control method is best for organizations, and is not well suited for smart home IoT applications [40,43].
C. Discretionary access control (DAC) is an access control where permissions are granted based on the identification of what group/entity a user belongs to. DAC uses an access-control list that contains information on users’ permissions. This list is consulted to identify which users do and do not have access to certain resource objects. This type of access control is easier to implement, needing just a simple username and password. When compared to MAC, DAC has fewer restrictions, and is considered to be less secure. This is because specific access control is already enforced for all subjects and objects within an information system by a security administrator [43]. DAC is less secure in the sense that anyone given access can make changes to resource objects, and the security administrator can see who is granted access or access controls [2]. As security is of the utmost importance in many IoT applications, this method will not be considered for implementation in this blockchain environment.
D. The rule-based access-control (RBAC) technique is known to be a preventive approach, because it allows the system owner to customize and personalize the type of access that one can have to view resources. The kind of access a person has is based on their role within an entity. RBAC adopts predefined roles in a system, and different access levels are granted based on these roles [18,30]. Access controls are set based on what “group” a user falls into, according to their role and responsibilities within the organization. Preset criteria allow a user to have certain permissions, to view certain resources and systems [40]. Maintaining an RBAC system that covers all possible scenarios can be complex and difficult to manage in a smart home blockchain environment. Furthermore, having pre-defined rules limits the flexibility of the system, making it difficult to adapt new devices to the network. For this reason, RBAC is not suitable for a blockchain smart home in the IoT [33].

### 4.3. Attribute-Based Access Control—A Suitable Blockchain Access-Control Method

Now that different access-control techniques have been explored, and found to be unfavorable methods for blockchain IoT applications, attribute-based access control (ABAC) will be presented as a suitable access-control method. This access-control method will be explained in detail, along with how it can be integrated into the Ethereum and Hyperledger Fabric platforms. Understanding the details behind ABAC and how they can be integrated into these two popular platforms is crucial for a fair and comprehensive comparison of their capabilities.

A blockchain implements ABAC when decisions for access are made by analyzing the attributes of the subjects and resources of the environment or context. ABAC is beneficial due to its nature of providing fine-grained and flexible access controls for numerous characteristics in decisions. ABAC includes intricate policies, and is able to provide effective access-control decisions, because it concerns environmental attributes in decision making [44].

**Figure 4:**
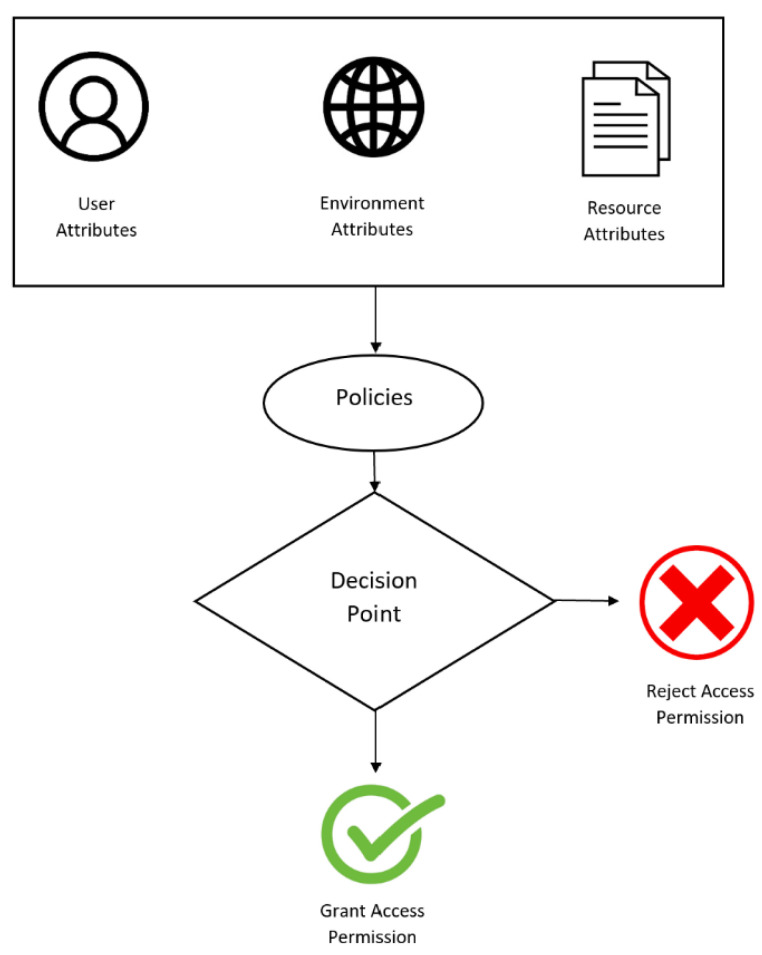
Blockchain-based access-control application.

Figure 4 shows a general diagram of the methodology for ABAC. There are three attributes with the subject: user-based, environment-based, and resource-based attributes. These attributes are passed through, while the policies in place are also considered. The system will then determine if the policies will allow for the object to be accepted or rejected, based on the attributes given [35].

**Figure 5:**
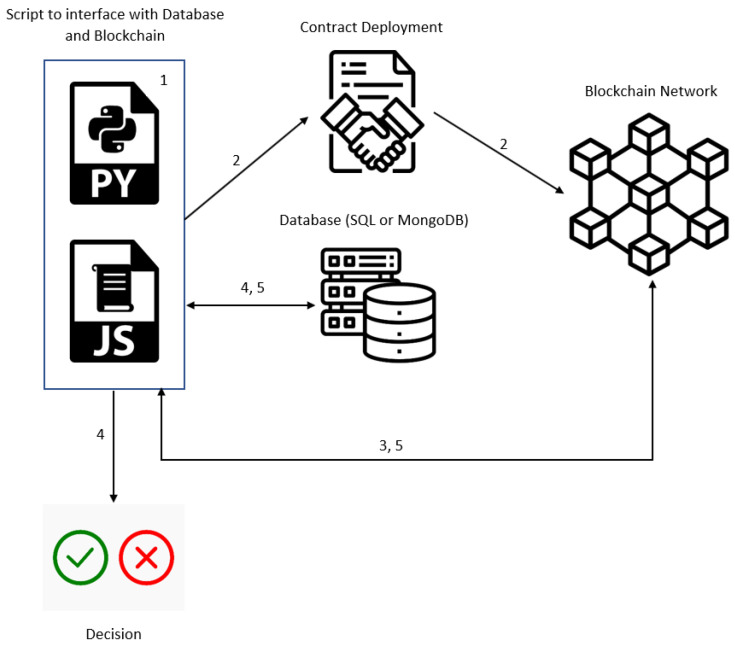
ABAC with blockchain implementation.

A blockchain holds various features that allow for ideal access-control systems that have immutability and transparency. When a malicious administrator alters a policy to deny or allow access to a user, the event will be supervised and documented by the blockchain network. This is because traces of updates affiliated with the blockchain cannot be wiped [44]. A general diagram of how the blockchain can interact with the ABAC system has been developed (Figure 5). The flow of the system starts with the Python or JavaScript code that interacts with the blockchain and the database used. When a contract is deployed, a new instance is created on the blockchain network. This will go back to the script to check the ABAC policies, to determine if permissions should be granted or rejected. During this process, the database allows the data to be read, written to, updated, or deleted, as long as permission is granted. After this is done, the blockchain network is then updated.

The blockchain platform Hyperledger Fabric has the capability to adopt ABAC, to create a more secure network [45]. Hyperledger Fabric integrates ABAC, and implements a modular structure that includes a membership layer. This membership layer can authenticate users, and grant access to users, based on their access level and system policy. The integration of ABAC in Hyperledger makes it possible to build permission groups for access control, by examining member attributes [44]. The blockchain platform Hyperledger, within the IoT domain, has an access-control system based on ABAC [37].

An Ethereum blockchain platform adopts ABAC through the use of smart contracts [45] on an existing network. Access requests can be made on the network using ETH mined on the private network. This request will follow ABAC security characteristics, and can either be granted or denied, similar to how ABAC is used in Hyperledger Fabric. As both the Hyperledger Fabric and Ethereum blockchain networks can implement ABAC, it makes sense to compare these two systems in an IoT environment [46,47].

### 4.4. Blockchain Access Control: Smart Contract Access Control

This section aims to provide an overview of how smart contracts can be used for access control in a blockchain network. As the majority of this paper will discuss the use of smart contracts, it is important to understand their capabilities and their relationship with access-control implementation, to conduct a fair comparison.

A smart contract is a self-executing contract, with terms and conditions, between a buyer and a seller. These two parties are explicitly written into lines of code contained in the smart contract. Smart contracts are constantly being implemented within blockchain networks, and have numerous access controls for IoT systems. IoT access-control systems are based on common access-control functions, examples of which are the RBAC and ABAC models [48]. As mentioned earlier, RBAC access control is based on the role (i.e., administrative, guest) of figures within an organization [28,30]. Access rights, such as read, write, and execute, are assigned to subject roles. RBAC is configured so that many-to-many relationships can be established between the access rights and the subjects [48]. ABAC is based on policies. These policies involve various types of attributes, such as object, subject, and environmental attributes (i.e., the organization’s resources). Attributes within ABAC-based schemes define various rules and policies, stating under what conditions access rights can be granted to subjects [49,50].

### 4.5. Access-Control Framework with IoT Environment

In order to effectively compare Ethereum and Hyperledger Fabric in the context of IoT access control, it is essential to have a clear understanding of the different components that make up an IoT system. By understanding the role and functions of the many components, such as servers, storage devices, and IoT gateways, the complexities and challenges that come with managing access control in such a system can be better appreciated. This also allows the identification of access-control requirements, which can then inform the evaluation of the suitability of Ethereum and Hyperledger Fabric for ABAC in the IoT.

There are various approaches to constructing an IoT system; however, generally, the system should comprise servers, storage apparatus, IoT gateways, and user devices that are interconnected via a peer-to-peer (P2P) network [19]. In addition, an IoT system should incorporate IoT devices, such as sensors, actuators, software, and other related components.

By interacting with the IoT and storage devices within the system, a server enables services to be performed on the network [51]. For example, the server can collect data from the sensors, to gather information about the system environment. It can also tell actuators on the network to perform an action, or even send data to the storage devices to create a database of information [19]. This is crucial for an IoT system, because it allows the necessary tasks to be performed; otherwise, the system would be useless.

Storage devices allow data to be stored on the system. A variety of data can come from any of the peers on the network, such as servers, sensors, and users [19]. This allows the network to identify trends, and optimize performance for a more efficient network. It is also important to store the network’s history, in case of an emergency, or for security reasons.

User devices are any devices on the network that a user can interact with to access the services on the network [19], such as checking the temperature, turning on the lights, and unlocking/locking [16] a door in a smart home. The devices on the network are typical devices that people interact with daily, such as laptops, smartphones, or other personal computers that have software to control these network functions.

An IoT gateway connects IoT devices (such as temperature sensors and motion detectors) in the network, using a common short-range wireless communication interface [19], such as Bluetooth Low Energy (BLE) and Wi-Fi. A common interface is used to ensure that devices can communicate in a cohesive network. For example, a cluster of sensors may be connected utilizing Bluetooth technology, so that a user can interact with the sensor using a user device on the network [52].

In an IoT system, the server plays a crucial role in collecting data from sensors, to obtain information about the environment. Additionally, it can send commands to actuators, and store data on storage devices [16] to create a database of information. The storage devices can receive data from any peer on the network, such as sensors, servers, or users, allowing a diverse range of data to be stored. User devices are any devices that a user can interact with to access services, such as laptops, smartphones, or other personal computers. An IoT gateway connects IoT devices on the network, using Bluetooth, Wi-Fi, and other short-range wireless communication technologies. For example, a cluster of sensors may be connected together using Bluetooth technology, enabling users to interact with them using their user devices. An IoT device is designed to collect data about the environment and transmit them to a server or storage device [8]. Generally, these devices are either sensors or actuators, capable of gathering information or performing specific tasks within the network. For instance, a sensor may monitor the temperature of the surroundings, while an actuator can adjust the heating or cooling of the environment [53,54].

Included in a reliable access-control framework within an IoT environment is the smart contract platform. As mentioned before, a smart contract can be implemented on an Ethereum platform, which will be briefly described, in order to highlight its important elements [55].

The first element of the network is the account/address, which identifies the account in the network. Many blockchain platforms have two account types, for externally controlled accounts and contract accounts [56] (also known as smart contracts). These are unique addresses that are used to identify and modify the functionality of the network.

The next is the smart contract which, as mentioned above is an account that has a function and data. Once the smart contract is written, it is pushed onto the blockchain in a platform-specific bytecode format. The function of the smart contract is vast, and can even be used for application binary interfaces (ABIs) [19]. Smart contracts can be interacted with by sending a transaction from one account to the smart contract’s account, sending a message from another account, or using a call function to modify the data within the smart contract [57,58].

Finally, mining is a crucial process in the Ethereum platform, as it involves generating new blocks that can be used for transactions. To do this, miners repeatedly guess random numbers to solve a cryptographic problem. When they solve the problem, they send the block to other nodes on the network for validation. If the block passes validation, it is added to the blockchain network but, if it fails, it is discarded. This creates a reliable and secure method of adding new blocks to the network, making it difficult to add invalid blocks. This is particularly important for developing a secure access-control system in an IoT network [18,58,59].

An access-control contract (ACC) is created and deployed by a peer on the IoT network, to manage and restrict access requests made by another peer on the network, known as the subject. The peer who deploys the ACC is referred to as the object. While a single pair of object and subject peers can have multiple ACC-governing requests, each ACC can only be linked with one object-and-subject pair [60]. ACC policies consider several parameters, such as the resource, action, permission, and time of the last request. The resource refers to the file or storage unit where the policy is located, while the action is the activity that will be performed on the resource. The permission is predefined to either allow or deny the action, and the time of the last request indicates the last time that a request was made [61]. When there is a violation of the ACC policy, it is considered a misbehavior, and the subject, time, and penalty of the action are considered and recorded.

## 5. Experimentation

In this section, the results of our experimental investigation into ABAC in a smart home environment using a blockchain will be presented. The data in this section were obtained through observations on the Ethereum and Hyperledger network platforms [62]. The data were collected and analyzed using the methods previously discussed. The following subsections present a detailed analysis and comparison of the experimental data.

### 5.1. Experiment Setup

In the following subsubsections, a detailed overview of the experimental setups for both the Ethereum and Hyperledger fabric networks will be provided. These setups serve as the foundation for this comparison in evaluating the effectiveness of the ABAC mechanism in blockchain systems.

The first Section 5.1.1, delves into the configuration and components of the Ethereum network. It includes a description of the hardware specifications of the laptop and Raspberry Pi, as well as the software used to build the Ethereum network. Furthermore, an explanation is provided to show how the ABAC smart contract is initialized using Truffle, to offer a comprehensive understanding of this crucial component.

In the next Section 5.1.2, the setup for the Hyperledger Fabric network, within a virtual machine running on a Dell XPA 13 9300, is presented. The advantages of using a virtual machine are discussed, along with the modifications made to the chain code to test and compare the ABAC aspect of this network.

By establishing these two experimental setups, a solid groundwork is established for the following experimental analysis. A table is provided at the end of this subsection, to serve as a summary of the hardware used in each network (Table 3).

#### 5.1.1. Ethereum Network Setup

The Ethereum private network was built using one laptop (Microsoft Surface Book 2) and one microprocessor (Raspberry Pi 3 Model B). The laptop is running Windows 11 Pro, with a 2-core Intel Core i7-8650U CPU, with a base frequency rated at 1.90 GHz, and 16 GB of RAM. The Raspberry Pi 3 Model B is running the Raspberry Pi OS Lite operating system, with a quad-core ARM Cortex A53 processor at 1.2 GHz, and 1 GB SDRAM. The laptop is running the bulk of the private Ethereum network, with two miners, and the Raspberry Pi is an IoT node on the network.

On both devices, the Geth client was installed, to transform the devices into Ethereum nodes. This installation allows the user to seamlessly interact with the Ethereum network, granting the user the ability to perform activities, such as creating accounts, mining ETH, and deploying smart contracts. The Geth client serves as a crucial tool in facilitating these important operations within the Ethereum environment.

On the laptop, two miners were created and synchronized [63], to provide the ETH necessary to perform actions on the network. Miner #1 has four total accounts and Miner #2 has two accounts that can be used to send and receive transactions. The miners only need a minimum of one account; however, more were created to verify the synchronization, and for testing purposes. The miners were synchronized via the creation of a static-node.json file. This file was also saved to the other trusted nodes on the network, such as the Raspberry Pi. On the Raspberry Pi, two accounts were made to store any gas sent from other accounts on the network. These accounts were also synchronized with the miners, to complete the network [47].

The Ethereum smart contract was initialized using Truffle, a popular tool for blockchain smart contract development. Through the use of Truffle, the smart contract can be built, compiled, and migrated onto the private Ethereum network. The smart contract, written in the Solidity programming language, integrates ABAC into the network. Once migrated, a JavaScript file is executed, to enable the interaction between the smart contract and the blockchain network [47].

The developed ABAC smart contract considers a user’s admin status, age, and ban count number. The admin status will determine if the account has administrative purposes or not. The age will ensure that no one under the age of 18 is trying to access the network. The ban count keeps track of the number of bans the user has. This number must be less than three for the user to have access to the network. All three of these attributes will ensure that a user has proper permission to access the Ethereum network, and any IoT devices connected to the system. The usage of this smart contract will be explained in detail in the next experimentation section.

#### 5.1.2. Hyperledger Fabric Setup

The Hyperledger Fabric is running on a Dell XPS 13 9300 within a virtual machine. The virtual machine or VM is being run on Oracle VM VirtualBox Managers, with the operating system being Ubuntu version 22.04.01. Running the Hyperledger Fabric network on a VM offers the advantage of saving at different points, to prevent losing all progress [64,65,66].

The test network available on the Hyperledger Fabric network was used to evaluate the ABAC portion of the Hyperledger Fabric network. The chain code is also based on a sample that is available on the official Hyperledger Fabric GitHub page. There were some modifications made, so that two attributes were needed to utilize the functions enabled by the chain code, with one attribute needed for adding assets to the network, and the other attribute to update the added assets [46].

A table has been created to serve as a comprehensive summary of all the device hardware and software specifications (Table 3).

### 5.2. Results Comparison

#### 5.2.1. Ethereum ABAC Implementation

**Figure 6:**
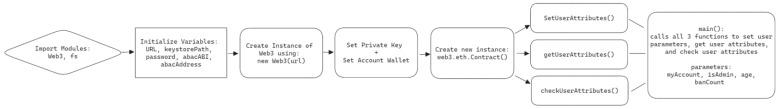
Ethereum ABAC contract interaction flowchart.

**Figure A1:**
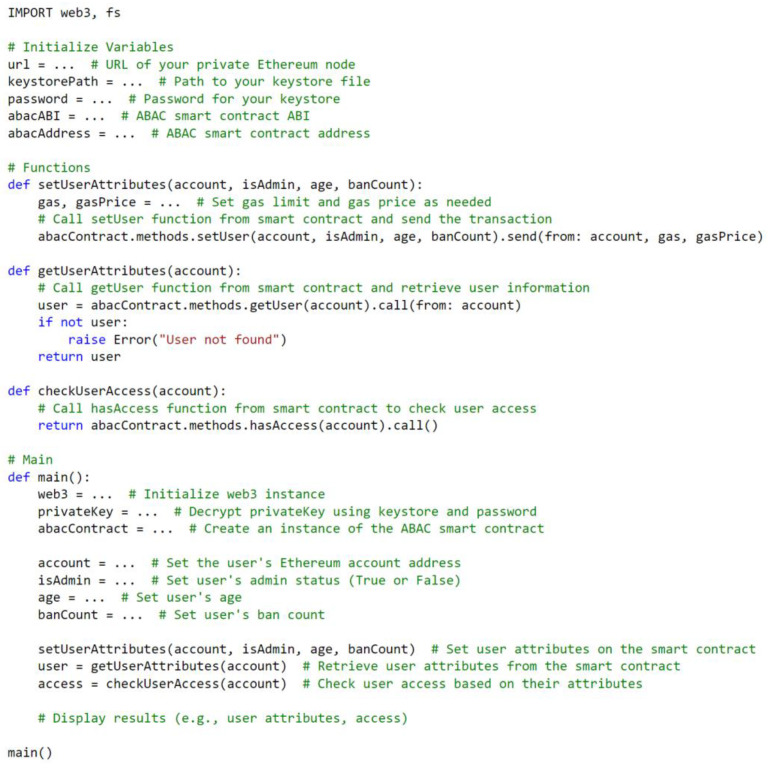
Ethereum ABAC smart contract pseudo code, with comments.

To implement ABAC into Ethereum, it is necessary to develop a smart contract, to be migrated onto the network. An original smart contract was developed, without the use of any existing code or template, to create the ABAC framework for comparison. Algorithm 1 presents the methodology used to write the code for the smart contract designed in the experiment. The code initializes some variables, including the URL of the Ethereum node, the keystore path, the password to the Ethereum node, the application binary interface (ABI) of the smart contract, and the address of the smart contract. Next, three functions are defined: *setUserAttrivutes()*, *getUserAttributes()*, *checkUserAccess()*, and *main()*. The functionality of each of these functions will be explained in more detail below; however, these functions set the attributes of the user, display the attributes of any user on the network, and check whether the user has access to the network or not. Figure 6 shows a simplified version of the discussed algorithm, in the form of a flowchart. In addition to the algorithm and flowchart, pseudo code has also been provided in the appendix of this paper, to provide another example of how this smart contract has been implemented (Figure A1).
**Algorithm 1:** Ethereum ABAC Contract InteractionImport modules: Web3, fsInitialize Variables:a.Set *URL* variable to private Ethereum nodeb.Set *keystorePath* to path of local keystore filec.Set *password* to keystore passwordd.Set *abacABI* to smart contract ABI after migratione.Set *abacAddress* to smart contract address after migrationf.Read keystore file and store in *keystore*
Create new instance of Web3 with *new Web3 (url)*Set private key from the keystore using *password*Set the account wallet to be used for sending transactionsCreate a new instance of the ABAC smart contract using the function *web3.eth.Contract()* with ABI and addressDefine *setUserAttributes():*a.Has parameters: *myAccount*, *isAdmin*, *age*, *banCount*, *callback*.b.Set variables *gas* and *gasPrice* as per required.c.Call *setUser* method of the ABAC smart contract with the provided parameters and send the transaction.d.Pass the *myAccount*, *gas*, *gasPrice*, and *callback* to the send function
Define *getUserAttributes():*a.Has parameters: *myAccount*, *callback*.b.Call *getUser* method of the ABAC smart contract with the provided account.c.Pass the *myAccount* and *callback* to the call function.d.If there are no errors and the user has valid attributes (*isAdmin*, *age*, *banCount*), invoke the callback with null and the user object. e.Otherwise, invoke the *callback* with an error indicating the user was not found.
Define *checkUserAccess():*a.Has parameters: *myAccount*, *callback*.b.Call *hasAccess* method of the ABAC smart contract with the provided account.c.Pass *myAccount* and *callback* to the call function.
Define main():a.Get the list of accounts.b.Retrieve the first account from the list.c.Get the network ID.d.Set values for parameters *myAccount*, *isAdmin*, *age*, and *banCount.*e.Call *setUserAttributes()* with provided parameters.f.If there are no errors, log the transaction hash.g.Call *getUserAttributes()* to fetch the defined user attributes.h.If there are no errors, log the user attributes.i.Call the *checkUserAccess()* to check user access.j.If there are no errors, log if the user is granted access or not
Call main() to initiate the contract interaction

**Figure 7:**
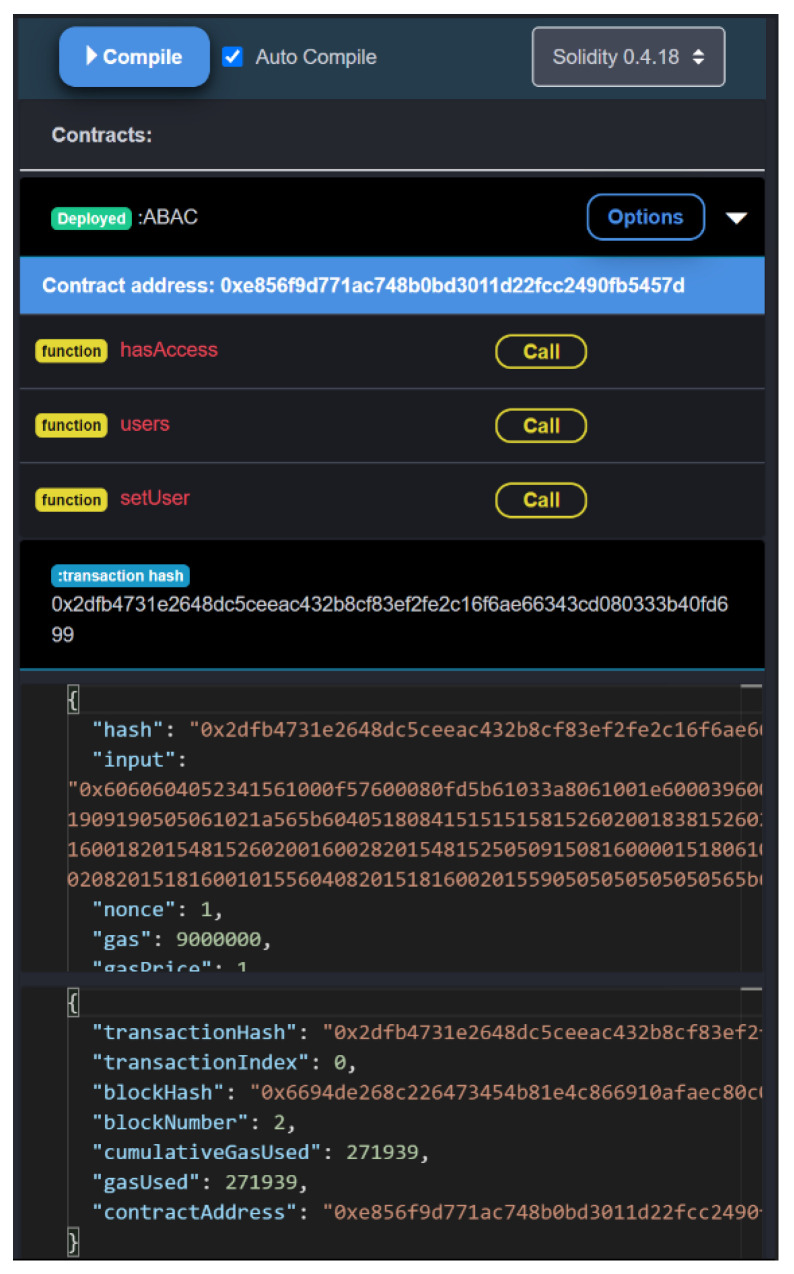
ABAC smart contract deployed.

The smart contract was tested using EthFiddle, an open-sourced blockchain browser application that developers can use to help construct their blockchain network. Here, the ABAC smart contract was imported and deployed, to test the results of implementing the contract (Figure 7). The gas was set at 9,000,000, to ensure no transactions were pending. The wallet address, which is the same as the user address, was also generated, to receive any transactions to the wallet.

**Figure 8:**
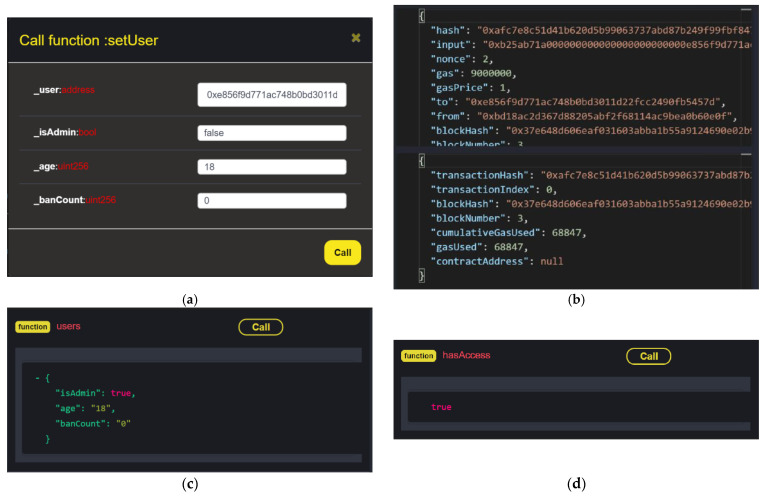
(**a**) ABAC smart contract deployed with *setUser*; (**b**) transactions on the blockchain network; (**c**) display user attributes with *users*; (**d**) using the *hasAccess* function to display the user-access status.

After running the contract, the results show that the contract successfully implements ABAC based on the set attributes. To create a new user on the network, the function setUser is called. This is where the users’ attributes are set, by defining the user address, Boolean admin status, age, and the number of times the user has been banned. In the case shown in Figure 8a, the user address is set, the user is not an admin, the user is 18 years old and, as it is a new account, the ban number is zero. After all the attributes are set, a transaction hash is generated, to confirm that the new account has been validated and added to the blockchain (Figure 8b). The cost for making a new account takes a gas price of one, which is relatively small compared to the 9,000,000 gas initialized. This value is also small compared to a blockchain network with two synchronized miners.

The user function can also be called to display the account that is currently part of the network. Here, the user address must be specified, to ensure the information is secure. This prompts the specified user attributes to be displayed in the terminal (Figure 8c), indicating their admin status and age, and the number of times they have been banned. This can be used to keep track of the users on the blockchain network, and to ensure that users have the correct defined attributes.

The last interactive function in the smart contract is hasAccess, which determines whether or not an account should have access to the network resources. For example, Figure 8d shows a user checking the access status of a user with the address 0xe856f9d771ac748b0bd3011d22fcc2490fb5457d. As the function returned true, this user has the correct attributes to access the blockchain network. If this were to be false, the user would not have access to the network, and would have to wait until their attributes changed, or would never have access to the network resources.

The ABAC smart contract has proven to be a successful and effective implementation of attribute-based access control on a blockchain network [21,67]. Through testing, the results show that a user can be added to the network, with their attributes defined, and their access status determined by the *hasAccess* function [32]. The cost of making a new account is small compared to the gas on the network, indicating that the contract is efficient in terms of gas consumption. The user function can also be called to display the attribute information of a specified account, to ensure the users have the correct defined attributes, and to see if the correct users are on the network. Overall, the ABAC smart contract provides a secure and reliable way to manage access to resources on an Ethereum network, based on the predefined attributes a user has.

#### 5.2.2. Hyperledger Fabric ABAC Implementation

**Figure 9:**
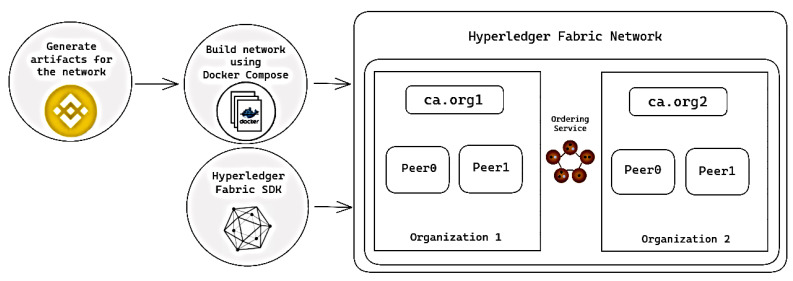
Hyperledger fabric network architecture.

ABAC was also implemented using Hyperledger Fabric, to demonstrate ABAC in a Hyperledger environment. Hyperledger Fabric was used as the framework for the network, due to its popularity and wide variety of resources. The smart contract was based on a pre-existing contract, with some modifications. The test network was chosen to be used as a guide for the network, due to its simplicity and the relative ease of setup. The network consists of two peer organizations and an ordering organization, which, in this case, consists of a single-node Raft ordering service. In addition, all of this is set up in an isolated docker-compose environment, making it impossible to connect to other fabric nodes that are running (Figure 9). In a full network, there can be many more organizations, a TLS Certificate authority, and the ability to connect with other nodes.

**Figure 10:**
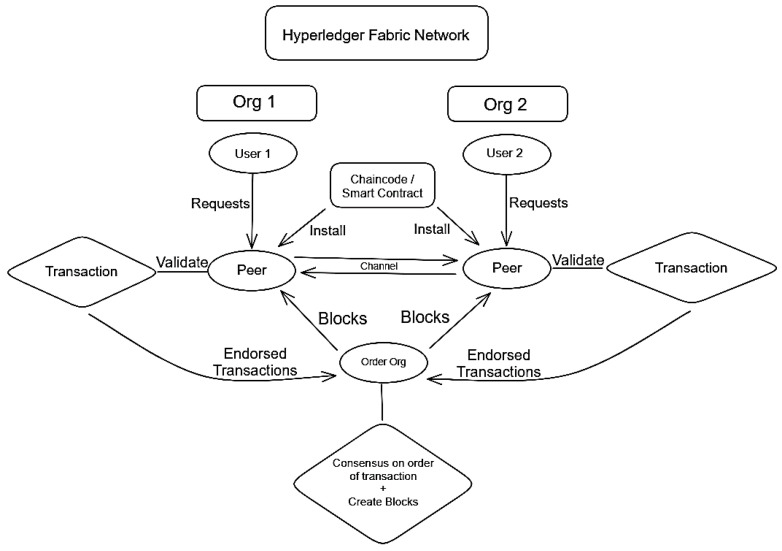
Hyperledger fabric network flow.

While the network is simplified, it still functions in the same way that a full network would. Figure 10 demonstrates the full network flow, starting with a user initiating a request to a peer. The peer, which has a chain code installed, will invoke the chain code, and check to see if the user’s request is valid, based on what is permitted in the chain code. If it is validated by the peer, the transaction will become “endorsed”, which means that the peer has signed it with its private key. This endorsed transaction is sent to the orderer node, which will come to a consensus on the order of transactions, and put that information into blocks. It should be noted that a network will typically have multiple orderer nodes but, in ours, there is only one, to simplify transactions. Once the blocks have been made, they will be sent to all peers on the network, which will then store them on a copy of the ledger [68].

**Figure 11:**
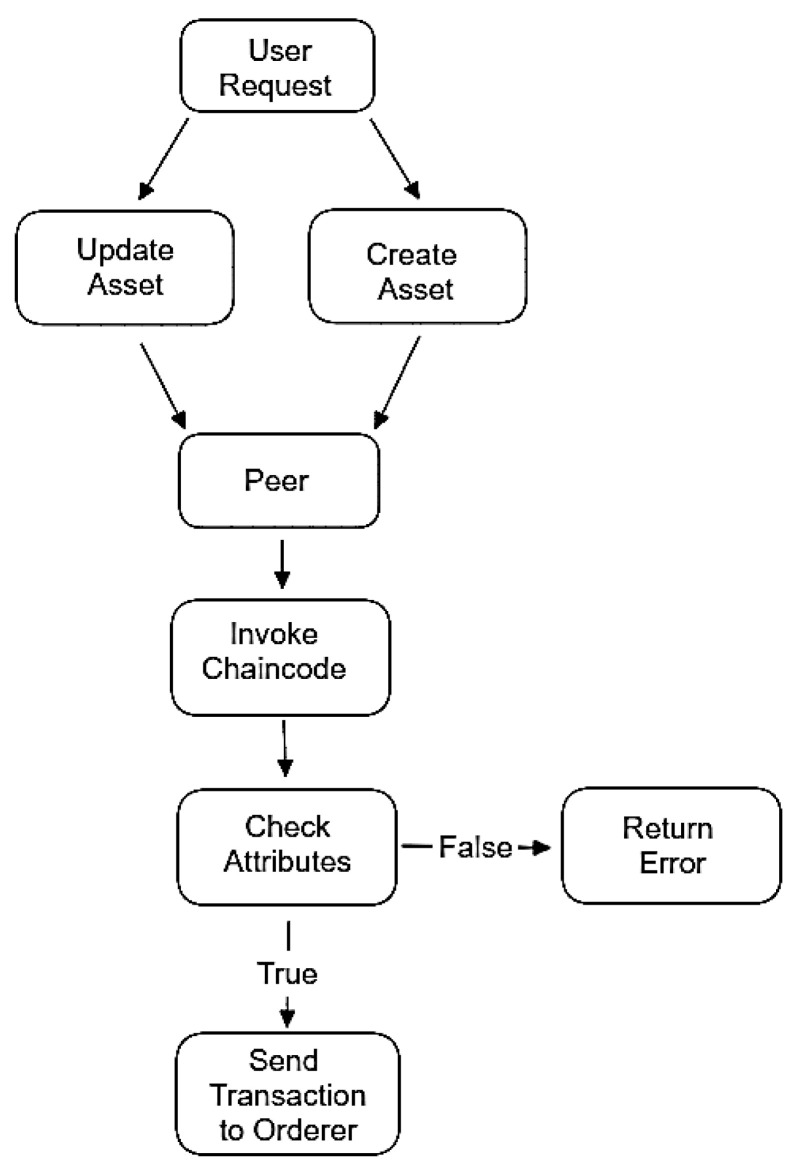
Hyperledger ABAC.

While the peers and orders validate and create blocks, it is the chain code that dictates what is valid and invalid. The chain code is installed on all the peers, and acts as the smart contract for the peer. The peer will check any transactions against the requirements stated on the chain code, before validating and endorsing transactions [69]. In our chain code, we have two requirements that have to be met. The user must have the “admin” attribute in order to create assets, and the user must have the “family” attribute to update assets. The peers will check for one of the requirements based on whether it is a request to add assets, or a request to update assets, while Algorithm 2 demonstrates how the Hyperledger Fabric ABAC works in a simplified formula (Figure 11).
**Algorithm 2:** Hyperledger Fabric ABAC Contract InteractionSet up Hyperledger Fabric network and deploy chaincodeInitialize Variables:a.Generate certificate for identity with *Attribute 1(A1)* and *Attribute 2(A2)*b.Enroll identity
Define *CreateAsset*a.Define Asset parameters: name, status, length, widthb.Call *GetClientIdentity()*. *AssertAttributeValue* function of the ABAC smart contract.c.Checks to see if the User has the abac.family attributei.If *A1*|*A2* == *abac.family* --> Create Assetii.If *A1*|*A2* ≠ *abac.family* --> Access denied message

*Define UpdateAsset*a.Define Asset parameters: name, status, length, widthb.Call *GetClientIdentity()*. *AssertAttributeValue* function of the ABAC smart contract.c.Checks to see if the User has the abac.Admin attributei.If *A1*|*A2* == *abac.Admin* --> Update Assetii.If *A1*|*A2* ≠ *abac.Admin* --> Access denied message
Check all assets with *GetAllAsset* or individually with *ReadAsset* with the name parameter

**Figure 12:**
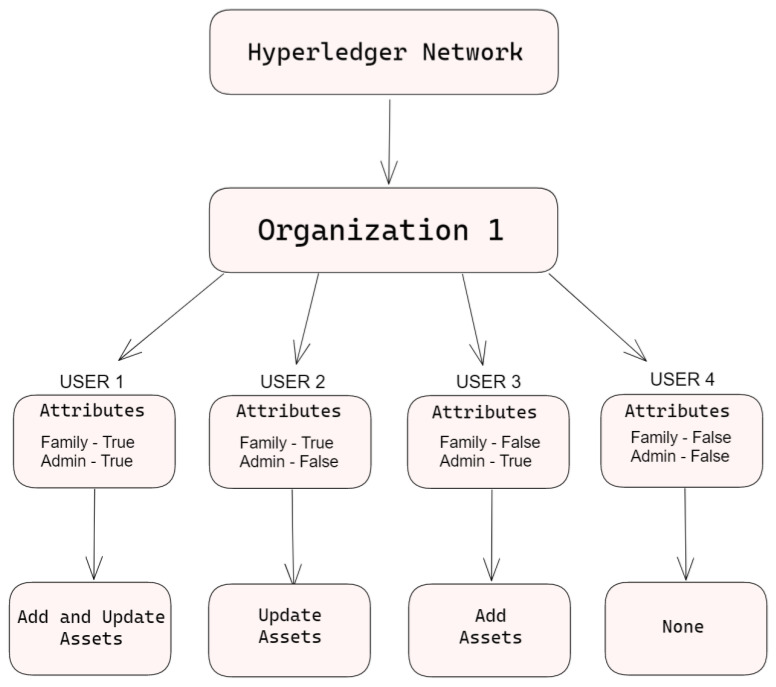
Hyperledger Fabric: users and attributes.

In our demonstration, the use of ABAC is presented by creating four users that have a different combination of two attributes. All the attributes of the users are displayed in Figure 12 below.

**Figure 13:**
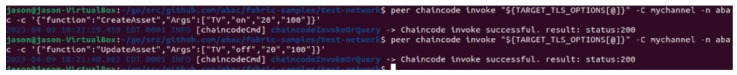
Hyperledger Fabric: user 1.

The first user is created with the admin and family attribute, which allows them to create assets or “IoT devices” on the network, as well as update those created assets, such as turning them “on” or “off”. This is shown when they successfully invoke the chain code for both actions, and get a result status of 200 (Figure 13), indicating that it was successful [70].

**Figure 14:**
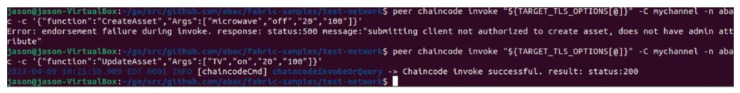
Hyperledger Fabric: user 2.

Another user, user 2, has the family attribute and a notAdmin attribute. While we could just leave out the admin attribute, the notAdmin attribute makes it easier to visualize which attribute is true. This combination of attributes allows user 2 to update existing assets on the network, such as turning something “on”, but when they try to create an asset, they will receive a status 500, as well as a message telling them that they are lacking the admin attribute (Figure 14).

**Figure 15:**
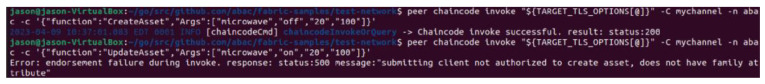
Hyperledger Fabric: user 3.

A third user has the admin attribute but not the family attribute, which means that they are able to add assets on the network, but are unable to update assets once they are already on the network. If they attempt to do so, they will receive a status 500, as well as a message telling them that they are lacking the family attribute and do not have access (Figure 15).

**Figure 16:**
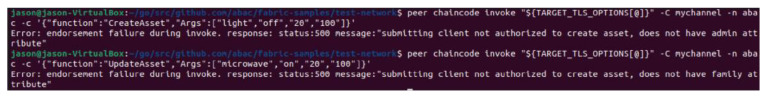
Hyperledger Fabric: user 4.

Finally, user 4 has neither the admin nor family attribute, which means that they are not able to invoke any valid transactions on the network (Figure 16). They will receive a status 500 for all attempted requests.

**Figure 17:**
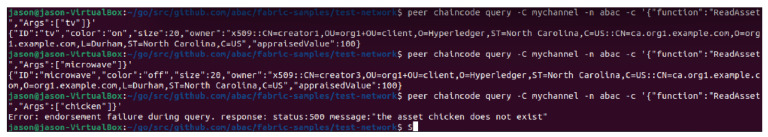
Querying individual assets.

**Figure 18:**
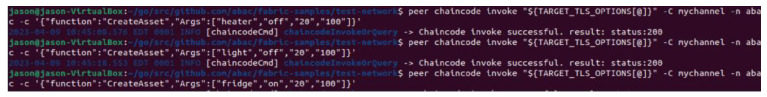
Creating more assets.

**Figure 19:**
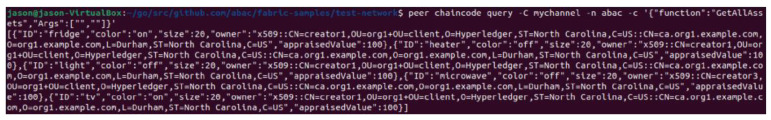
Querying all assets.

In addition to the creation and updating of assets, users are capable of querying the network, to see what assets are available, and what state they are currently in. This is stored in a world state, which only keeps track of the most up-to-date versions of the asset, as opposed to the ledger, which keeps track of all the assets states, and any transactions made to update or create them. Figure 17 demonstrates this on individual assets, where we have two valid assets that exist, and one that is invalid and does not exist. The “tv” and “microwave” assets exist on the network, allowing us to get all the information on them, while the “chicken” asset does not exist, resulting in a status 500. Figure 18 shows user 1 creating three more assets, “heater”, “light”, and “fridge”. Figure 19 shows all the assets on the world state in one query, in alphabetical order from A-Z. All the information displayed is from the most up-to-date version of the assets.

## 6. Discussion and Analysis

This section presents a detailed discussion and analysis of the research findings, focusing on a novel comparative evaluation between Ethereum and Hyperledger Fabric for the implementation of ABAC within an IoT environment. The objective is to assess the advantages and limitations of these two prominent blockchain platforms in relation to ABAC, using an original Ethereum smart contract and a modified Hyperledger Fabric chain code for a fair comparison. Throughout the experimental process, the time required to construct the blockchain network with ABAC, the cost of implementing the ABAC code, and the support for a smart home environment are to be evaluated in this comparison. The subsequent subsections provide a detailed analysis of these factors. By examining these key factors, this comparison aims to provide decision-makers with valuable insight to guide their selection of the most suitable blockchain platform for ABAC implementation in an IoT environment. The table presented at the end of this section (Table 4) presents a summarized comparison of Ethereum and Hyperledger Fabric for ABAC implementation in a smart home IoT environment, highlighting their respective strengths and weaknesses, based on the provided experimental analysis.

### 6.1. Comparison between Ethereum and Hyperledger Fabric ABAC Implementation Results

ABAC implementation and deployment are both areas where Ethereum and Hyperledger Fabric have demonstrated themselves to be strong platforms. However, a novel comparison between these two popular platforms, in regard of time, cost, and support for a smart home IoT, can be used to highlight the benefits and shortcomings of using these platforms in an ABAC IoT environment [70]. The subsubsections below will analyze these previously unexplored topics, for comparison between Ethereum and Hyperledger Fabric in this setting.

### 6.2. Time to Build Blockchain Network with ABAC

The support that Ethereum has for smart contracts allows flexibility when creating an ABAC environment; however, the time required to construct an Ethereum blockchain network and develop a smart contract is significantly longer than that of Hyperledger Fabric. The Ethereum network must be built from scratch, for there are currently no test networks available to download. For example, all nodes and accounts built on the Ethereum network, such as the miners and the inclusion of the Raspberry Pi, were made from the ground up. This introduces more points for possible errors, which take time to remedy. Only after the network is successfully built can a smart contract be deployed onto the network and, in the case of the ABAC smart contract used in this experiment, the solidity contract had to be fully developed [71].

Although it takes more time to build an Ethereum network and ABAC smart contract for an IoT environment, this allows for more customizability from the start, as each step of the process is made by the user, with their specific needs in mind.

Hyperledger Fabric makes it simple to build and implement features into a blockchain environment, with the test networks available. The chain code can be more easily found and, in this case, the existing chain code was modified for the purposes of this experiment, instead of starting from the ground up. This makes implementation much faster, and is good for cases where ABAC has to be quickly implemented into an IoT environment, for experimental purposes or real-world environments [72].

### 6.3. Cost of Implementing ABAC Code

Another comparison when implementing ABAC into Ethereum and Hyperledger Fabric concerns the gas consumption that is required to run transactions and execute smart contracts on Ethereum [34]. Gas is an important component of the Ethereum network, due to its public nature, ensuring transactions are accounted for. However, this consumption of gas requires miners to be running on the network at all times, to ensure no transactions are left pending, and the smart contract can be migrated onto the network without any problems. The inclusion of miners requires more processing power and, ultimately, more strain on the system components. For example, while running the Ethereum network, a considerable strain was placed on the laptop running the network, due to the mining that was taking place.

As Hyperledger Fabric is completely private, there is no gas required for the implementation of the chain code. The cost of implementing the ABAC chain code is not considered, making it easier to interact with the network without concern about the cost. This also allows for lower-powered systems to be running the network, without the concern of mining [73].

### 6.4. Support for Smart Home Environment

Although not yet performed, the experimental procedure showed that the support and ease of implementing physical IoT devices into the network are higher than those for Hyperledger Fabric [74]. The inclusion of the Raspberry Pi in the network makes it simple to connect sensors, measuring devices, smart lights, and other equipment to the network. Once the ABAC smart contract is migrated to the network, user access to these physical components can be limited, based on their attributes. The support for including physical IoT devices to a Hyperledger Fabric has been the subject of limited research, and seems to be less supported, making Ethereum a better option for implementing ABAC into a smart environment.

## 7. Implication of Future Research

In the future, there are several areas of study that necessitate exploration in the context of implementing ABAC within IoT environments, using blockchain technology. Expanding the experimentation to incorporate a broader range of IoT devices would provide information on the performance and parameters of both the Ethereum and Hyperledger Fabric networks. This would enable a better understanding of the scalability, resource management capabilities, and device support, while enhancing the practicality for real-world IoT deployments.

Additionally, it is also important to conduct testing, to evaluate the resilience of the ABAC system against blockchain-specific attacks [75]. By performing simulated blockchain attacks, researchers can identify potential weaknesses, and develop countermeasures, to ensure that the integrity of the ABAC system is not compromised [34].

Furthermore, a comparative analysis between Ethereum and other IoT-specific Hyperledger blockchain platforms, such as Hyperledger Sawtooth and Hyperledger Iroha, would be beneficial. By expanding the comparison to other platforms, researchers can gain a deeper insight into the unique features available, allowing decision-makers to make informed decisions regarding the most suitable blockchain platform for their ABAC IoT network.

Through the realization of future research plans, advancements can be made in the field of blockchain-based ABAC in IoT environments. This ultimately contributes to developing a more secure, scalable, and efficient access-control system for the inevitable expansion of blockchain-based IoT environments.

## 8. Conclusions

Presented in this paper is a comparison of ABAC implementation into the widely used Ethereum and Hyperledger Fabric blockchain platforms, for an IoT smart home environment. A comprehensive summary of access-control and blockchain-specific methods, the development of a new ABAC Ethereum smart contract, and a modification of an existing Hyperledger Fabric smart contract allowed for a conclusive comparison to be made. Ethereum and Hyperledger Fabric have demonstrated their strengths in ABAC implementation and deployment. Ethereum’s support for smart contracts allows for a more flexible environment when creating a blockchain network with ABAC, but the time required to construct this Ethereum blockchain network, and develop a smart contract, is significantly longer than that of Hyperledger Fabric. Hyperledger Fabric, with the availability of test networks and chain code, makes for a significantly faster implementation process. Additionally, the requirement for gas on the Ethereum network makes the inclusion of miners essential to the network [76]. Hyperledger Fabric eliminates this requirement, making the network more suitable for systems with a low processing power. However, Ethereum provides more support for including physical IoT devices in the network, making it a more practical network to build for an ABAC environment [2]. Hyperledger Fabric’s support for such devices is limited, and makes it difficult to support an environment for physical IoT applications, with the growing popularity of smart homes and other smart environments.

## Figures and Tables

**Table 2:** Comparison of traditional access-control methods for blockchain IoT environments.

Traditional Access-Control Method	Description	Reason Method Is Not Suitable for Blockchain IoT
Mandatory access control (MAC)	Highly secure method that restricts user access based on predefined security labels	Time-consuming setup and not ideal for many blockchain IoT applications
Role-based access control (RBAC)	Determines authorization based on user’s job, responsibilities, and authority level in an organization	Hierarchical nature limits the flexibility needed for dynamic and decentralized blockchain IoT environments
Discretionary access control (DAC)	Utilizes access-control lists to define user’s permissions for accessing specific resources	Its simple implementation raises security concerns in applications where data integrity is crucial
Rule-based access control (RBAC)	Access based on predefined roles and levels	Complex-to-manage rule sets, making it challenging to adopt in a dynamic blockchain IoT environment

**Table 3:** Summary of hardware and operating systems.

Device	CPU	Operating System	Memory	Hard Disk	Platform
Microsoft Surface Book 2	Intel Core i7-8650U 4.2 GHz max	Windows 11 Pro (64-bit)	16 GB	512 GB	Ethereum
Dell XPS 13 9300	Intel Core i7-1065G7 3.9 GHz max	Windows 11 Home	16 GB	460 GB	Hyperledger Fabric
Raspberry Pi 3 Model B	Quad-core ARM Cortex A53, 1.2 GHz	Raspberry Pi OS Lite	1 GB SDRAM	16 GB (microSD card)	Ethereum

**Table 4:** Comparison of Ethereum and Hyperledger Fabric for ABAC implementation in an IoT environment based on the analysis of results.

Aspect of Comparison	Ethereum	Hyperledger Fabric
Time to build blockchain network with ABAC	Longer construction time for network and smart contract development due to no pre-existing test networks or full solidity contracts	Faster implementation using existing test networks and modified chain code
Cost of implementing ABAC code	Gas generation and consumption for transactions and smart contract execution, resulting in higher system demands	No gas consumption, leading to lower system strain and easier interaction with network
Support for smart home environment	Higher support for connecting physical IoT devices to Ethereum network; easier integration of smart equipment	Limited support for adding physical devices to this platform; not the ideal platform for a smart home environment

## Data Availability

No new data were created or analyzed in this study. Data sharing is not applicable to this article.
